# aMatReader: Importing adjacency matrices via Cytoscape Automation

**DOI:** 10.12688/f1000research.15146.2

**Published:** 2018-08-10

**Authors:** Brett Settle, David Otasek, John H Morris, Barry Demchak

**Affiliations:** 1 Department of Medicine, University of California, San Diego, California, 92093-0688, USA; 2 University of California San Francisco, San Francisco, California, 94143, USA

**Keywords:** Workflow, Reproducibility, Cytoscape, Interoperability, REST, Microservice, Adjacency

## Abstract

Adjacency matrices are useful for storing pairwise interaction data, such as correlations between gene pairs in a pathway or similarities between genes and conditions. The aMatReader app enables users to import one or multiple adjacency matrix files into Cytoscape, where each file represents an edge attribute in a network. Our goal was to import the diverse adjacency matrix formats produced by existing scripts and libraries written in R, MATLAB, and Python, and facilitate importing that data into Cytoscape. To accelerate the import process, aMatReader attempts to predict matrix import parameters by analyzing the first two lines of the file. We also exposed CyREST endpoints to allow researchers to import network matrix data directly into Cytoscape from their language of choice. Many analysis tools deal with networks in the form of an adjacency matrix, and exposing the aMatReader API to automation users enables scripts to transfer those networks directly into Cytoscape with little effort.

## Introduction

Adjacency matrices are a strong choice for storing pairwise element interaction data, such as those commonly produced by biological analysis tools to represent a weighted network of relationships between biological components (such as genes, conditions, pathways, times, etc.). aMatReader facilitates importing general adjacency matrices (such as correlation, similarity, and difference data) into edge attributes of Cytoscape networks. aMatReader aims to enable users to compile Cytoscape networks from one or multiple matrix files by creating edges or edge attributes for nonzero values in the matrix. 

We upgraded the original aMatReader ^1^ to enable Cytoscape Automation ^2^ by exposing two new REST endpoints ^3, 4^, bridging the gap between network matrix data in automation scripts and Cytoscape. With Cytoscape Automation, biologists can manipulate Cytoscape networks via REST calls and create complex workflows in their language of choice (e.g. Python and R). Researchers can then utilize Cytoscape’s filtering tools to remove redundant or unremarkable edges between components, slimming the network and emphasizing stronger relationships to further their analysis.

In this paper, the Implementation section describes the general approach of aMatReader and its REST endpoints. The Operation section describes how to call the endpoint as a Cytoscape Automation Function. The Use Case section demonstrates how to import adjacency matrices into Cytoscape via the aMatReader endpoint, and the Discussion section describes the import performance.

**Figure 1.**
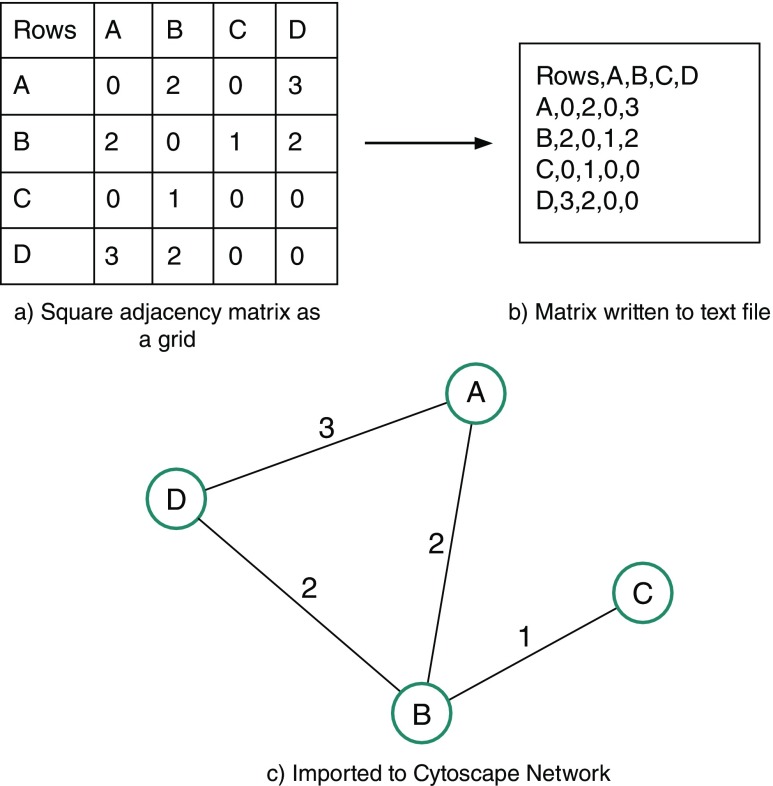
Converting a square adjacency matrix into an undirected network. ( **a**) In Excel, Python, etc., the matrix is stored as a 2-dimensional array with optional labels. ( **b**) The matrix is exported to a comma delimited file. ( **c**) Importing the undirected matrix into Cytoscape with aMatReader, edges are defined by nonzero values within the upper triangle.

aMatReader translates adjacency matrices into Cytoscape networks by adding edges or edge attributes represented by non-null values in the matrix. The square adjacency matrix is the standard matrix representation of a network. In a square matrix, node labels are stored in the first row and column of a table of size (N+1, N+1). The *N*× *N* grid of values within the table contains edge weights. A non-null value at cell *(i, j)* represents the weight of an edge between node *i* and node *j*. An example matrix text file and graph representation can be seen in Figure 1.

If an analysis tool is calculating distance or similarity between all pairs of genes within pathways, it will likely produce a square adjacency matrix. Some other examples of square matrices are covariance and correlation matrices. Square matrices are often symmetric, meaning row and column names are similarly ordered, the value at cell *(i, j)* is the same as that at *(j, i),* and the values along the diagonal are calculated by comparing an element to itself. A symmetric matrix represents an undirected network, and only refers to the upper triangle for edge attributes.

In the case of diagonal square matrices, it can be efficient to omit row names, because the row name at index *i* is identical to the *i* ^th^ column name. This is especially useful when exporting matrices from Python using numpy by inserting node names to the file as a header row. (ref Listing 1 numpy example)

However, there are many cases where an adjacency matrix is not square. For example, a correlation matrix between genes and conditions will have different row elements than column elements. The network generated by such a correlation matrix will be directed and bipartite. As a directed network, the entire matrix is used to generate edge attributes, unlike undirected networks that only use the upper triangle.

## Methods

### Implementation

aMatReader is developed to handle a wide spectrum of adjacency matrix formats. For directed network matrices, row names represent “source” nodes and column names represent “target” nodes. To accommodate possible missing or reordered row and column names, we use an adjacency list of node indices as an intermediary data structure. Two separate arrays are used to store source and target node names, where the *i* ^th^ name in the array refers to the node at index *i* in the adjacency list. Once the matrix has been translated, aMatReader makes a pass through the adjacency list and set edge attributes in the network.

One constraint of aMatReader is that the parser expects integer or floating point values. Any String, Boolean, or unrecognized values will be considered null and no edge will be created (and no warning will be generated).

There are two possible options for importing an adjacency matrix into a Cytoscape network. To create a new network from an adjacency matrix file, the caller can use the **import** Function. If the adjacency matrix defines edge attributes that should be added to an existing network, the **extend** Function should be used. This is especially useful because an adjacency matrix can only represent one type of edge attribute, and complex networks are often represented by multiple adjacency matrix files.

If a new network is being created (via the **import** Function, described in the Operation section below), all of the network nodes are created and named first. Then each non-null value in the matrix is used to create an edge. The edge attribute takes its name from the name of the matrix file that is being imported. 

If a network is being extended (via the **extend** Function, described in the Operation section below), aMatReader attempts to match row and column names to existing nodes in the network. If no node exists with the given name, a new one is created. Creating edge attributes is handled similarly; an edge between the source and target node is added if it does not already exist, and then the attribute is set.

 Some matrix formats add extra information to provide insight to the parser. Matrices produced by cCrepe and MATLAB optionally prefix column names with a period-delimited description of the weights specified by the matrix (e.g. “sim.score” or “q.value” in cCrepe). Additionally, comments can be included in files by inserting a hash symbol at the start of a line. Below is an example gene similarity table produced by MATLAB. More examples of supported format idiosyncrasies can be seen in the documentation provided on the Cytoscape App Store.

# With row and column names, the cell at (0, 0) is ignored
# PIPE delimited adjacency matrix, with column name prefix "distance."
Rows|distance.nodeA|distance.nodeB|distance.nodeC|...
nodeA|0|2.0|1.45|...
...

**Listing 1.** **Sample adjacency matrix with confusing format. Pipe “|” delimited text file with comments and column name prefix.**

### Operation

To use aMatReader from the Cytoscape Desktop interface, the user has a few options. They can click the “Import Matrix Files” menu action under Apps>aMatReader. A file chooser dialog will appear allowing the user to select one or multiple files to be imported. Alternatively, the user can also can drag and drop matrix files (renamed to have a .adj extension, if necessary) into the Cytoscape Network panel. Once files have been identified, the import parameters (as listed below) will be predicted by parsing the first few lines of one of the files. A second dialog will appear enabling the user to view and manipulate the parameters before attempting to import all files simultaneously. The intended audience of the rest of this paper is workflow programmers hoping to automate the import of adjacency matrices into Cytoscape.

**Figure 2.**
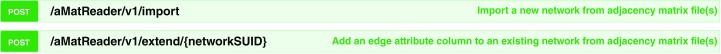
Functions exposed by the aMatReader API, as documented in Swagger.

aMatReader exposes two Functions ^5^ via the Cytoscape CyREST API, **import** to create a new network and **extend** to add edge attributes to an existing network. If necessary, the caller specifies the network to be extended as part of the REST URL. The Function endpoints ( Figure 2) enable users to manipulate network data as internal Cytoscape data, and are documented in the **Apps: aMatReader** section of the Swagger document available via **Help → Automation → CyREST API**.

Both endpoints expect the same parameters within the JSON body of the request:

{
  "files": ["list", "of", "paths"],
  "delimiter": "TAB",
  "undirected": false,
  "ignoreZeros": true,
  "interactionName": "interacts with",
  "rowNames": true,
  "columnNames": true,
  "removeColumnPrefix": true
}

The **files** parameter specifies a list of local file paths for matrix files, and is the only required parameter; all other parameters default to the values shown above. Files imported in the same REST call must have the same format and thus be importable with identical parameters. The caller can specify the matrix **delimiter** (as one of “PIPE”, “SPACE”, “TAB”, or “COMMA”), whether the matrix is symmetric and diagonal and should only import the upper triangle as **undirected**, whether or not to create edges for zero values (called **ignoreZeros**), edge interaction type (called **interactionName**). The payload can also define whether row and column names are present in the file (called **rowNames** and **columnNames**). The **removeColumnPrefix** parameter informs the parser to ignore a common prefix in column names, if it exists.

Note that the interaction type is only set for edges created by the import, and is not set for pre-existing edges in an **extend** call.

The aMatReader endpoints return a CIResponse ^6^ according to Cytoscape Automation best practices. If the call succeeds, the CIResponse contains an import result object (as the **data** element); otherwise, it contains an explanation of the error (as the **errors** element):

{
  "data": {"newEdges": integer,
           "updatedEdges": integer},
  "errors": []
}

 The **newEdges** value contains the number of edges created in the network, and the **updatedEdges** contains the number of edges that already existed and received new edge attribute(s).

If the delimiter is unrecognized or any of the matrix files cannot be found or fails to parse correctly, the **errors[0].status** element returns 404, and the remainder of the **errors[0]** element contains additional information.

In order to download and use aMatReader, ensure that you are running Cytoscape version 3.6.0 or later with at least 512MB of free memory to store the matrix before creating the edges.

***Calling aMatReader Functions.*** To import files to a new network, the caller must send an HTTP POST request to **/v1/aMatReader/import** with a JSON payload object specifying the list of matrix files and any optional parameters listed above. To extend an existing network, the caller must also pass the **networkSUID** parameter as part of the URL ( **/v1/aMatReader/extend/{networkSUID}**).

Note that the **networkSUID** must be an integer. The caller can determine a network’s SUID via the / **v1/networks** endpoint.

Example code is provided in R, Python and as a Bash curl, but can easily be adapted into any language that supports REST calls.

**R**

# Basic settings for cyREST
port.number = 1234
base.url = paste("http://localhost:", toString(port.number), "/v1", sep="")

# Send it to Cytoscape!
amatreader.url = paste(base.url, "aMatReader", "import", sep="/")
amatreader.args = list(files="path", delimiter="TAB", undirected=FALSE, ignoreZeros=TRUE, interactionName="correlates to", rowNames=TRUE, columnNames=TRUE, removeColumnPrefix=FALSE)
amatreader.JSON = toJSON(amatreader.args)
res <- PUT(url=amatreader.url, body=amatreader.JSON, encode="json")

**Python**

import requests, json
resp = requests.put("localhost:1234/v1/aMatReader/import", data=json.dumps(data))
resp = resp.json()

**Bash**

curl -X PUT --header 'Content-Type: application/json' --header 'Accept: application/json' -d '{ \ 
  "files": [ \ 
     "string" \ 
   ], \ 
   "delimiter": "TAB", \ 
   "undirected": false, \ 
   "ignoreZeros": true, \ 
   "interactionName": "interacts with", \ 
   "rowNames": true, \ 
   "columnNames": true, \ 
   "removeColumnPrefix": true \
 }' 'http://localhost:1234/v1/aMatReader/import'

## Use cases

 The simple use case that inspired an upgrade to the original aMatReader app was filtering correlation data in search of similarities among different stages of disease severity. The R package cCrepe gives compositionally corrected scores for all pairwise connections in a dataset, producing an adjacency matrix for similarity score and q-values. Both files contained row names, as well as column names prefixed by “sim.score” or “q.value”, respectively. aMatReader allows the user to import both files into one Cytoscape network, which can easily be filtered with a few extra calls to the core Cytoscape CyREST API (as shown below):

**Python**


import requests, os, json
CyREST = "http://localhost:1234/"

headers = {
  'Content-Type': 'application/json',
  'Accept': 'application/json'
}
paths = [
    os.path.join(os.getcwd(), "q_values.csv"),
     os.path.join(os.getcwd(), "sim_scores.csv")
]

data = {
  'files': paths,
  'delimiter': 'TAB',
  'undirected': True,
  'ignoreZeros': True,
  'interactionName': 'interacts with',
  'rowNames': False,
  'columnNames': True,
  'removeColumnPrefix': True
}

# Execute post, import matrix files to a single network
res = requests.post("http://localhost:1234/aMatReader/v1/import", data = json.dumps(data), headers=HEADERS)
res = res.json()
suid = res['data']['suid']

# perform filtering via Cytoscape Automation
edges = requests.get('http://localhost:1234/v1/networks/{SUID}/edges'.format(SUID=suid))
edges = edges.json()

# loop through edge SUIDs and remove it if the q_val is below a certain threshold
# To get edge attributes, use:
for edge in edges:
    attrs = requests.get('http://localhost:1234/v1/networks/{networkSUID}/tables/defaultedge/rows/{edgeSUID}'.format(networkSUID=suid, edgeSUID=edge), headers=HEADERS)
    attrs = attrs.json()
    if attrs['q_values.csv'] < .5:
        requests.delete('http://localhost:1234/v1/networks/{SUID}/edges/{edgeSUID}'.format(SUID=suid, edgeSUID=edge), headers=HEADERS)

The response to the aMatReader function call will create a Cytoscape network with edges that each have a **sim_score** and **q_val** column. With a few extra lines, the script can perform filtering and analysis without any interaction from the user. Exposing the aMatReader API enables the researcher to completely automate their analysis process without leaving their script.

## Discussion

aMatReader parses files line by line, composing an intermediate weighted adjacency list to cleanly handle the multiple supported matrix formats. The most difficult case is a matrix that provides node names in the first column (instead of the first row) – this requires discovering the node names by parsing the adjacency matrix file line by line until it reaches the end of the file. The intermediate adjacency list solves this problem by converting the matrix to a more iterable data structure. Unfortunately, this also causes a small performance and memory hit because the second data structure takes O(E) memory and must be iterated over again. To avoid this case, we recommend that the user arrange for the adjacency matrix files to be an edge table (or SIF file), which Cytoscape core can easily understand. A possible future improvement to the algorithm could be made by generating a new network while iterating through the matrix, then merging that network with the desired target network.

### Future plans

 aMatReader was designed with existing adjacency matrix exporters in mind, such as the igraph.Graph.Adjacency ^7^ and pandas DataFrame ^8^ objects. We will continue to improve aMatReader to handle more diverse matrix formats, including string matrices, and to provide a CyREST Function that returns the predicted parameters for importing a matrix file.

## Summary

In this paper, we present aMatReader, a general adjacency matrix importer app for Cytoscape 3. aMatReader creates another data entry method that allows researchers to join multiple adjacency matrices, each representing an edge attribute, into one network. aMatReader was developed to handle importing diverse matrix file formats into Cytoscape via automation scripts and libraries.

The aMatReader CyREST API is composed of two import Functions that enable users to create a new network or extend an existing network with an adjacency matrix defining edge attributes. Users can also import multiple adjacency matrices that represent different edge attributes in the same network simultaneously before calling Cytoscape’s advanced filtering functions.

## Data availability

The data referenced by this article are under copyright with the following copyright statement: Copyright: © 2018 Settle B et al.

Data associated with the article are available under the terms of the Creative Commons Zero "No rights reserved" data waiver (CC0 1.0 Public domain dedication). 

All data underlying the results are available as part of the article and no additional source data are required.

## Software availability

**The aMatReader app is available on the Cytoscape App Store:** http://apps.cytoscape.org/apps/aMatReader.

**Source code available from:** https://github.com/idekerlab/aMatReader.

**Archived source code available from:** https://doi.org/10.5281/zenodo.1287303 ^9^.

**License:** GNU Lesser General Public License v2.1.
