# Supplementary material for: aMatReader: Importing adjacency matrices via Cytoscape Automation
Source: F1000Res. 2018 Aug 10;7:ISCB Comm J-823. Originally published 2018 Jun 21. [Version 2] doi: 10.12688/f1000research.15146.2 (PMC6039939; doi:10.12688/f1000research.15146.2)
Supplement: Peer review history

# Peer review: Referee response for version 2

**DOI:** 10.5256/f1000research.17328.r36983

**Published:** 2018-08-10

**Authors:** Kimberly Glass

**Affiliations:** 1 Department of Medicine, Harvard Medical School, Boston, MA, USA

no additional comments

I have read this submission. I believe that I have an appropriate level of expertise to confirm that it is of an acceptable scientific standard.

# Peer review: Referee response for version 1

**DOI:** 10.5256/f1000research.16500.r35307

**Published:** 2018-07-03

**Authors:** Kimberly Glass

**Affiliations:** 1 Department of Medicine, Harvard Medical School, Boston, MA, USA

this is a clearly-written summary of the updated aMatReader API. This tool will be useful for reading in certain types of networks (e.g. correlation networks) into Cytoscape.

 A few additional thoughts:

- A description of how the tool works from within Cytoscape would be a nice addition to the paper for the less-computationally-savvy users of Cytoscape.
- It would be useful for the authors to clarify that for directed networks, rows in the input file represent "source" nodes and columns "target" nodes.
- I found the discussion section too short. Additional discussion on how aMatReader overcomes the limitation described, and/or when there is an advantage to the matrix format over an adjacency list representation would be useful.

I have read this submission. I believe that I have an appropriate level of expertise to confirm that it is of an acceptable scientific standard.

# Response

**Published:** 2018-08-02

**Authors:** Brett Settle

**Affiliations:** UCSD Ideker Lab, USA

We would like to thank the referee for the positive feedback and respond to the very helpful comments:

- We wrote this publication as part of a collection of Cytoscape Automation papers, which focus on enabling researchers to programmatically access CyREST endpoints exposed by apps, over Cytoscape Desktop access.  However, I agree that it would be useful to include a section for non-programmers near the beginning.
- Thank you for noticing this omission. We will add the row/column information to the paper.
- We will be sure to add more information to the discussion section in the near future.  The current implementation of aMatReader translates the matrix to a mapping to better handle sparse matrices. Cytoscape core network importer supports adjacency lists so that format would be preferred, but some workflows output one or many adjacency matrices that need to be imported into Cytoscape all at once.

# Peer review: Referee response for version 1

**DOI:** 10.5256/f1000research.16500.r35308

**Published:** 2018-07-02

**Authors:** Hideo Matsuda

**Affiliations:** 1 Graduate School of Information Science and Technology, Osaka University, Osaka, Japan

This paper presents a tool for importing adjacency matrices into Cytoscape. The tool is particularly useful to represent weighted biological networks, such as co-expression relationships of genes, confidence scores of predicted relationships, etc.

 Minor comment: In "files" parameter in the JSON code, the character codes of some double quotes (") are different from others.

I have read this submission. I believe that I have an appropriate level of expertise to confirm that it is of an acceptable scientific standard.

# Response

**Published:** 2018-08-02

**Authors:** Brett Settle

**Affiliations:** UCSD Ideker Lab, USA

We would like to thank the referee for positive feedback and helpful comments.

 The inconsistent character codes will be fixed.
